# Supplementary material for: Deep learning approach for screening neonatal cerebral lesions on ultrasound in China
Source: Nat Commun. 2025 Aug 20;16:7778. doi: 10.1038/s41467-025-63096-9 (PMC12368173; doi:10.1038/s41467-025-63096-9)
Supplement: Supplementary file 1 — Supplementary Information [file 41467_2025_63096_MOESM1_ESM.pdf]

Supplementary Information

Supplementary Table 1. Summary Statistics of Ultrasound Equipment Parameters. This table provides mean (standard deviation) values for parameters such as Mechanical Index (MI), Thermal Index (TIs), depth (X and Y, in cm), and probe frequencies (linear and curved, in MHz) across development, internal, external, and blind sweeping sets.

Supplementary Table 2. Statistical Summary of Ultrasound Device Manufacturers and Models. This table details the distribution of ultrasound devices by manufacturer and model across datasets.

Supplementary Tabel 3. Performance of Mid-Level Radiologists in Internal Test Set. The metrics include sensitivity (SEN), specificity (SPE), Positive Predictive Value (PPV), and Negative Predictive Value (NPV), each represented as median percentage (95% confidence interval, CI), with the 95% CI calculated using the Clopper-Pearson method. This table evaluates 11 mid-level radiologists (R1–R11) on an internal dataset

Supplementary Table 4. Performance of Mid-Level Radiologists in External Test Set. This table assesses the same 11 mid-level radiologists (R1–R11) on an external dataset.

Supplementary Table 5. Comparison of Junior Radiologist Group Without and With AI Assistance. This table compares the performance of nine junior radiologists (R1–R9) on internal and external test sets, with and without AI assistance. Metrics include SEN, SPE, and F1-score, reported as percentages. P-values between performances without and with AI assistance were calculated using a paired chi-square test.

Supplementary Table 6. Performance of NCLS in Normal vs. Abnormal classification. This table evaluates the NCLS for distinguishing normal versus abnormal cases across development, internal, and external test sets. Metrics include SEN, SPE, PPV, NPV, and F1-score, with multi-label classification applied.

Supplementary Table 7. Performance of NCLS in Normal vs. Mild vs. Severe. This table evaluates performance for normal, mild, and severe categories across development, internal, and external test sets. Metrics include SEN, SPE, PPV, NPV, F1-score, and AUC.

Supplementary Table 8. Ternary Classification Error Distribution. This table shows error distributions for mild conditions (Grade I-II IVH, ependymal cysts, ventricular dilatation) in internal and external test sets, reporting percentages misclassified as normal or severe.

Supplementary Table 9. Subgroup Analysis of Different Probe Types. This table compares performance using linear versus curved probes in internal and external test sets. Metrics include SEN, SPE, and F1-score, with P-values calculated by the Mann-Whitney U test.

Supplementary Table 10. Subgroup Analysis of Different Gestational Age Groups. This table analyzes performance across full-term, preterm, and very preterm GA groups in internal and external test sets. Metrics include SEN, SPE, and F1-score, with P-values calculated by the Kruskal-Wallis H test followed by Dunn's Multiple Comparison Test.

Supplementary Table 11. Assessment Outcomes Under Papile and Volpe Grading Systems. This table compares classification performance using Papile and Volpe grading systems across development, internal, and external test sets. Metrics include SEN, SPE, PPV, NPV, and F1-score.

Supplementary Table 12 Qualitative Scoring of Extracted Views by Senior Radiologists. This table presents qualitative scores (0–5 scale) by senior radiologists for AI-extracted views across four criteria: View Correctness, Detection Correctness, Anatomical Structure Recognition, and Clinical Value. View Correctness assesses alignment with optimal standard views (e.g., 5 = completely correct, matching the predefined standard plane perfectly). Detection Correctness evaluates structure detection accuracy, Anatomical Structure Recognition assesses clarity and completeness, and Clinical Value determines diagnostic utility.

Supplementary Table 13. Hyperparameter Search Result Table. This table lists the top-10 hyperparameter combinations ranked by a score (0.7 * SEN + 0.3 * F1-score).

Supplementary Table 14. Comparison Table of State-of-the-Art (SOTA) Models. This table compares performance of SOTA models including ResNet-50, ViT-Base, MambaOut, SigLipV2, MedViT-V2, MedMamba, Query2Label, and a baseline with enhancements (multi-label head, ensemble diagnosis). Metrics include SEN, SPE,

PPV, NPV, F1-score, and AUC.

Supplementary Figure 1. Distribution of Specific Conditions in the Dataset. This figure illustrates: (a) gestational age distribution (weeks, percentage), (b) probe type distribution (linear vs. curved), (c) brain injury severity classification (normal, mild, severe), and (d) disease subtype distribution across datasets.

Supplementary Figure 2. ROI annotated by the radiologist and the heatmaps. This figure displays original images, radiologist-annotated regions of interest (ROIs) for ventricles (red) and choroid plexus/bleeding foci (green) and heatmaps, highlighting areas of diagnostic focus.

Supplementary Figure 3. Reclassification of Original IVH Cases According to the Volpe Grading System. This figure includes: (a) Distribution of IVH grade changes in the development dataset, (b) IVH grade redistribution in the internal test set, (c) External validation of IVH reclassification. Each panel shows the redistribution of cases originally classified under the Papile grading (three columns representing IVH I-II, IVH-III, and IVH-IV, respectively) into the Volpe grading categories (normal, IVH-I, IVH-II, IVH-III, and PHI).

Supplementary Figure 4. Prognostic Outcomes Under Two Grading Systems.
This figure presents: (a) Prognostic outcomes under the Papile grading system, (b) Prognostic outcomes under the Volpe grading system.

Supplementary Figure 5. DBSCAN clusters in CUS videos. This figure visualizes the DBSCAN clustering results in coronal (a) and sagittal (b) plane videos. In panel a, the brown and green clusters represent the coronal plane slices of the third ventricle and the body of the lateral ventricle, respectively. These clusters assist in determining the scanning direction and filtering out irrelevant frames, such as those from the anterior horn (blue). In panel b, the red cluster corresponds to the mid-sagittal plane, while the purple clusters correspond to para-sagittal planes. The relative position of red and purple clusters helps to differentiate scanning directions and identify anatomical coverage in sagittal views.

Supplementary Figure 6. Anatomical structures detection and candidate frame scoring. This figure displays predicted bounding boxes over ultrasound images, with each label and confidence score corresponding to a detected anatomical structure. The prediction outputs serve as inputs to the candidate frame scoring pipeline. The scoring mechanism is further described in Supplementary Figure 7b.

Supplementary Figure 7. The workflow of the view extraction module. This figure illustrates the overall workflow for extracting standard views. (a) The architecture of the object detection module for anatomical structure localization. (b) The downstream candidate frame scoring process, which evaluates detected frames using anatomical structure confidence, DBSCAN-based cluster filtering, and positional scoring. Standard coronal views include COR1 (anterior horn), COR2 (third ventricle), and COR3 (body of the lateral ventricle).

Supplementary Figure 8. The architecture of the diagnostic model. This figure presents the multi-label diagnostic framework based on RegMixup-enhanced CNN features and multi-head self-attention aggregation. Six CUS images (i1–i6) are used as input for image-level prediction. TTA is applied both at the image-level and diagnosis-level. Final output includes multi-label classification and ensemble binary classification, indicating normal vs. abnormal findings.

Supplementary Table 1 | Summary Statistics of Ultrasound Equipment Parameters.

Set	Development set		Internal test set	External test set			Blind sweeping set
Subset	Train	Test		CS	SC	PY	
MI	1.17 (0.21)	1.15 (0.24)	1.28 (0.14)	0.87 (0.07)	0.89 (0.39)	0.96 (0.17)	1.37 (0.91)
TIs	1.09 (0.41)	1.09 (0.42)	1.24 (0.28)	0.64 (0.08)	0.19 (0.10)	0.19 (0.09)	0.97 (0.46)
Depth X (cm)	16.43 (4.15)	16.54 (4.44)	14.63 (4.78)	14.89 (0.98)	14.27 (1.25)	14.75 (1.25)	19.50 (2.52)
Depth Y (cm)	9.44 (2.42)	9.58 (2.58)	8.52 (2.64)	10.94 (0.45)	10.26 (0.69)	10.26 (0.69)	11.18 (2.16)
Linear (MHz)	9.32 (2.09)	8.96 (1.73)	9.17 (3.04)	8.71 (0.69)	8.52 (1.08)	8.40 (1.18)	7.550 (2.21)
Curved (MHz)	4.66 (1.17)	4.39 (1.27)	4.14 (1.42)	4.38 (1.17)	5.02 (0.97)	4.68 (1.12)	3.50 (1.09)

Mean (SD) values for Mechanical Index (MI), Thermal Index (TIs), depth (X and Y, cm), and probe frequencies (linear and curved, MHz) across development, internal, external, and blind sweeping sets. CS, SC, PY represent Changsha, Sichuan and Guangzhou Maternal and Child Health Hospitals, respectively.

Supplementary Table 2 | Statistical Summary of Ultrasound Device Manufacturers and Models

Set	Manufacturer	Model	Number	Manufacturer	Model	Number
Development set	GE Healthcare	LOGIQ S8	1044	GE Healthcare	LOGIQ E10	7
	GE Healthcare	LOGIQ E8	193	EDAN Instruments, Inc.	H8-12	10
	GE Healthcare	LOGIQ E9	82	GE Healthcare	Voluson E8	5
	EDAN Instruments, Inc.	Acclarix	86	GE Healthcare	Voluson E10	5
	Wisonic	Clivia 90	31	Mindray	Resona 7OB	46
Internal test set	GE Healthcare	LOGIQ S8	172	Philips	EPIQ Elite	1
	Toshiba	TUS-AI 900	16	Mindray	Resona 7	1
	GE Healthcare	LOGIQ E9	9			
External test set	Mindray	M9	117	Mindray	M11	130
	Siemens Healthineers	Sequoia	109			
Blind sweeping set	Toshiba	TUS-AI 900	47	GE Healthcare	LOGIQ E9	2
	GE Healthcare	LOGIQ S8	45	GE Healthcare	Voluson E10	2
	Philips	EPIQ Elite	6	Mindray	Resona 7	9

Supplementary Table 3| Performance of mid-level radiologists in internal test set.

	R1		R2		R3		R4	
	%	n/N	%	n/N	%	n/N	%	n/N
SEN	93.8	15/16	75.0	12/16	68.8	11/16	100.0	16/16
SPE	98.9	181/183	100.0	183/183	100.0	183/183	95.6	175/183
PPV	88.2	15/17	100.0	12/12	100.0	11/11	66.7	16/24
NPV	99.5	181/182	97.9	183/187	97.3	183/188	100.0	175/175
	R5		R6		R7		R8	
	%	n/N	%	n/N	%	n/N	%	n/N
SEN	87.5	14/16	100.0	16/16	68.8	11/16	81.3	13/16
SPE	98.4	180/183	96.2	176/184	100.0	183/183	98.9	181/183
PPV	82.4	14/17	69.6	16/23	100.0	11/11	86.7	13/15
NPV	98.9	180/182	100.0	176/176	97.3	183/188	98.4	181/184
	R9		R10		R11			
	%	n/N	%	n/N	%	n/N		
SEN	75.0	12/16	75.0	12/16	68.8	11/16		
SPE	98.9	181/183	98.9	181/183	99.5	182/183		
PPV	85.7	12/14	85.7	12/14	91.7	11/12		
NPV	97.8	181/185	97.8	181/185	97.3	182/187		

R, radiologist; SEN, sensitivity; SPE, specificity; PPV, positive predicted value; NPV, negative predicted value; %, Percentage;

Supplementary Table 4 | Performance of mid-level radiologist in external test set.

	R1		R2		R3		R4	
	%	n/N	%	n/N	%	n/N	%	n/N
SEN	88.5	23/26	61.5	16/26	38.5	10/26	73.1	19/26
SPE	96.1	317/330	100.0	330/330	98.9	326/330	93.6	309/330
PPV	63.9	23/36	100.0	16/16	71.4	10/14	47.5	19/40
NPV	99.1	317/320	97.1	330/340	95.3	326/342	97.8	309/316
	R5		R6		R7		R8	
	%	n/N	%	n/N	%	n/N	%	n/N
SEN	57.7	15/26	100.0	26/26	50.0	13/26	61.5	16/26
SPE	99.4	328/330	93.9	310/330	98.8	326/330	99.7	329/330
PPV	88.2	15/17	56.5	26/46	76.5	13/17	94.1	16/17
NPV	96.8	328/339	100.0	310/310	96.2	326/339	97.1	329/339
	R9		R10		R11			
	%	n/N	%	n/N	%	n/N		
SEN	84.6	22/26	80.8	21/26	92.3	24/26		
SPE	96.4	318/330	98.5	325/330	97.9	323/330		
PPV	64.7	22/34	80.8	21/26	77.4	24/31		
NPV	98.8	318/322	98.5	325/330	99.4	323/325		

R, radiologist; SEN, sensitivity; SPE, specificity; PPV, positive predicted value; NPV, negative predicted value; %, Percentage;

Supplementary Table 5 | Comparison of junior radiologist group without and with AI assistance.

Sets	Radiologist	SEN (%)		SPE (%)		AUC (%)		
		w/o. AI	w. AI	w/o. AI	w. AI	w/o. AI	w. AI	P-value
Internal test set	R1	100.0	100.0	84.2	87.4	91.3	93.8	0.0306
	R2	75.0	93.8	84.2	89.6	80.9	92.2	0.0110
	R3	100.0	100.0	85.8	91.3	92.5	95.5	0.0090
	R4	93.8	100.0	75.4	74.3	85.1	86.4	0.2459
	R5	93.8	100.0	94.0	89.6	95.6	94.3	1.0000
	R6	93.8	87.5	48.6	92.4	68.5	89.7	0.0003
	R7	81.3	100.0	96.7	92.4	90.4	95.9	0.1378
	R8	62.5	93.8	98.9	91.8	83.2	91.5	0.0632
	R9	87.5	100.0	98.4	95.6	96.0	97.3	0.3171
External test set	R1	84.6	92.3	94.2	93.0	88.9	92.4	0.1212
	R2	15.4	80.8	98.2	95.5	60.5	86.5	0.0000
	R3	76.9	84.6	96.7	93.6	83.5	87.0	0.1420
	R4	76.9	96.2	80.6	78.2	79.9	87.5	0.0223
	R5	88.5	92.3	95.2	92.1	92.8	92.0	1.0000
	R6	92.3	84.6	82.1	92.7	81.9	92.2	0.0253
	R7	88.5	96.2	92.4	92.1	90.6	93.7	0.1331
	R8	42.3	92.3	94.6	88.2	68.5	90.1	0.0001
	R9	88.5	92.3	97.3	95.8	92.4	93.5	0.2467

P values indicate AUC differences based on paired one-sided DeLong's test and were adjusted for multiple comparisons using the Benjamini–Hochberg false discovery rate correction. R, radiologist; SEN, sensitivity; SPE, specificity; AUC, area under curve; w/o. AI: Without AI assistance; w.AI: With AI assistance.

Supplementary Table 6 | Performance of NCLS in Normal vs. Abnormal classification.

Sets	SEN (%)	n/N	SPE (%)	n/N	PPV (%)	n/N	NPV (%)	n/N	F1-score(%)
Development test set	70.5	62/88	96.9	217/224	89.9	62/69	89.3	217/243	79.0
Internal test set	31.6	31/98	95.0	96/101	86.1	31/36	58.9	96/163	46.3
External test set	44.7	51/114	97.9	237/242	91.1	51/56	79.0	237/300	60.0

SEN, sensitivity; SPE, specificity; PPV, positive predicted value; NPV, negative predicted value; %, percentage;

Supplementary Table 7 | Performance of NCLS in Normal vs. Mild vs. Severe.

Set	Development test set			Internal test set			External test set		
Class	Normal	Mild	Severe	Normal	Mild	Severe	Normal	Mild	Severe
SEN	0.955	0.463	0.853	0.971	0.120	0.857	0.988	0.161	0.846
SPE	0.773	0.946	0.964	0.350	0.966	0.940	0.478	0.993	0.942
PPV	0.914	0.641	0.744	0.611	0.714	0.522	0.803	0.875	0.537
NPV	0.872	0.894	0.982	0.919	0.605	0.989	0.947	0.785	0.987
F1	0.934	0.538	0.794	0.750	0.206	0.650	0.886	0.272	0.657
AUC	0.922	0.842	0.971	0.680	0.624	0.939	0.832	0.754	0.959

SEN, sensitivity; SPE, specificity; PPV, positive predicted value; NPV, negative predicted value; F1, F1-Score %, percentage;

Supplementary Table 8 | Ternary Classification Error Distribution

Set	Category	Predict to Normal (N,%)	Predict to Severe (N,%)
Internal test set	Grade I-II IVH(n=34)	19 (46.34)	15 (36.59)
	Ependymal cyst(n=37)	37 (80.43)	0 (0.00)
	Dilatation(n=2)	1 (50.0)	1 (50.0)
External test set	Grade I-II IVH(n=62)	52 (77.61)	10 (14.93)
	Ependymal cyst(n=16)	16 (84.21)	0 (0.00)
	Dilatation(n=2)	2 (66.67)	0 (0.00)

The results are displayed in number (percentage) format, with the percentage indicating the rate of prediction errors among all cases for each disease type.

Supplementary Table 9 | Subgroup analysis of different probe type.

Dataset	Probe type	SEN	SPE	AUC	P-value
Internal test set	Linear	0.933(0.681-0.998)	0.925(0.875-0.959)	0.984	0.7004
	Curve	0.000(0.000-1.000)	0.900(0.555-0.998)	0.900	
External test set	Linear	0.938(0.698-0.998)	0.885(0.828-0.928)	0.981	0.7753
	Curve	0.900(0.555-0.998)	0.981(0.945-0.996)	0.969	

AUC values were compared between groups using the independent DeLong test for unpaired samples. P-values indicate whether the observed AUC differences are statistically significant (two-sided). SEN, sensitivity; SPE, specificity

Supplementary Table 10 | Subgroup analysis of different GA group.

Dataset	Internal test set			External test set		
Metric	SEN	SPE	AUC	SEN	SPE	AUC
Full-term	1.000 (0.158-1.000)	0.988 (0.936-0.999)	0.994	1.000 (0.158-1.000)	0.989 (0.959-0.999)	1.000
Preterm	0.600 (0.146-0.947)	0.921 (0.811-0.978)	0.961	0.500 (0.013-0.987)	0.964 (0.911-0.990)	0.982
Very preterm	1.000 (0.664-1.000)	0.781 (0.624-0.894)	0.989	0.933 (0.681-0.998)	0.559 (0.379-0.728)	0.859
P-value (Full-term vs. Preterm)	-	-	0.6460	-	-	0.7874
P-value (Full-term vs. Very preterm)	-	-	0.9135	-	-	0.0308
P-value (Preterm vs. Very preterm)	-	-	0.6688	-	-	0.1851

P-values represent pairwise AUC comparisons between gestational age groups, computed using the independent-sample DeLong test and adjusted for multiple comparisons using the Benjamini–Hochberg method. "-" indicates that no statistical significance test was performed for this metric. SEN, sensitivity; SPE, specificity

Supplementary Table 11 | Assessment Outcomes Under Papile and Volpe Grading Systems

**Test set**	**Classification system**	**SEN**	**SPE**	**PPV**	**NPV**	**F1-score**	**AUC**	**P-value**
Development test set	Papile	0.912	0.932	0.620	0.989	0.738	0.973	0.442
	Volpe	0.875	0.900	0.500	0.984	0.636	0.962	
Internal test set	Papile	0.875	0.934	0.539	0.988	0.667	0.981	0.748
	Volpe	0.833	0.939	0.577	0.983	0.682	0.981	
External test set	Papile	0.923	0.927	0.500	0.994	0.649	0.958	0.444
	Volpe	0.943	0.953	0.688	0.994	0.795	0.972	

The P-values indicate the statistical significance of differences in AUCs between gestational age groups, calculated using the independent-sample DeLong test (two-sided) and adjusted for multiple comparisons using the Benjamini–Hochberg method. SEN, sensitivity; SPE, specificity; PPV, positive predicted value; NPV, negative predicted value; F1, F1-Score.

Supplementary Table 12 | The qualitative scoring of the extracted views by senior radiologists.

Score	0(Completely wrong)	1(Mostly incorrect)	2(Minor errors)	3(Partially correct)	4(Mostly correct)	5(Completely correct)
View correctness	No frames match the standard plane.	Only 1-2 frames match, none are close to optimal.	3-4 frames match, none are optimal.	All frames match the standard plane, but none are optimal.	Frames are nearly optimal, with minor deviations.	The frames are the optimal and match the standard plane perfectly.
Detection correctness	No relevant structures detected, severe omissions.	Few structures detected, multiple key omissions.	Some structures detected, but many are inaccurate.	Most structures are detected, but a few are inaccurate.	Detection is mostly accurate, with minor errors.	All structures are correctly detected, with no omissions.
Anatomical structure recognition	No structures identified.	Most structures unclear or incorrect, with minimal recognition.	Some structures are clear, but the main ones are inaccurate.	Most structures are accurate, but a few are unclear.	Structures are mostly clear, with minor blur or missing details.	Structures are completely clear and accurate.
Clinical value	No diagnostic support; lesion area unclear.	Very limited diagnostic support; lesion area unclear.	Limited support; main structures unclear.	Good support, but some blurry areas and additional images needed.	Very helpful for diagnosis, with minor blurred areas.	Fully supports diagnosis with clear structures, no additional images needed.

Supplementary Table 13| Candidate frame scoring algorithm

Standard view	Candidate frame selection	Base score	Maximum appearance numbers
COR anterior horn view	Anterior horn of lateral ventricle	- Ant. horn lat. vent.: 0.2<br>- Corpus callosum: 0.3<br>- Vasal Ganglia: 0.15	- Ant. horn lat. Vent. :2<br>- Corpus callosum: 2<br>- Vasal Ganglia: 1
COR third ventricle view	The third ventricle	- Third ventricle: 0.4<br>- Ant. horn lat. Vent.: 0.1<br>- Corpus callosum: 0.3<br>- Vasal Ganglia: 0.1	- Ant. horn lat. Vent.: 2<br>- Corpus callosum: 2<br>- Vasal Ganglia: 1<br>- Third ventricle: 1
COR body view	Lateral ventricle	Lateral ventricle: 0.5	Lateral ventricle: 2
SAG midsagittal view	Two from:<br>- corpus callosum<br>- third ventricle<br>- cerebellum.	- Corpus callosum: 0.33<br>- Trigone of lat. vent.: 0.33<br>- Cerebellum: 0.33	- Corpus callosum: 1<br>- Trigone of lat. vent.: 1<br>- Cerebellum: 1
SAG left parasagittal view	Lateral ventricle or Vasal Ganglia	- Lateral ventricle: 0.3<br>- Vasal Ganglia: 0.7	- Lateral ventricle: 1<br>- Vasal Ganglia: 1
SAG right parasagittal view			

Each frame of a CUS video was processed by the object detection model and returned anatomical detection results. Candidate frame selection: If any of the anatomical structures were detected, the frame would be added to the candidate frame queue of the corresponding standard view. Base score: The total score of a frame was the sum of the base scores of all detected structures, multiplied by the corresponding prediction confidence. Maximum appearance number: To limit the situation that the same structure was detected multiple times and prevent inaccuracy scoring. Ant. horn lat. vent.: Anterior horn of lateral ventricle; Trigone of lat. vent.: Trigone of lateral ventricles

Supplementary Table 14| Hyperparameter search result table.

Batch size	Epochs	Image size	Learning rate	Sensitivity	Specificity	F1-score
16	45	256	1e-4	0.9394	0.9793	0.9118
16	45	128	1e-5	0.9394	0.9534	0.8493
16	25	256	1e-4	0.9091	0.9793	0.8955
16	25	256	1e-5	0.9091	0.9793	0.8955
16	25	128	1e-5	0.9394	0.9378	0.8158
8	45	128	1e-5	0.9091	0.9689	0.8696
8	25	256	1e-4	0.8788	0.9845	0.8923
16	45	256	1e-5	0.8788	0.9845	0.8923
16	60	256	1e-5	0.8788	0.9793	0.8788
8	25	256	1e-5	0.8788	0.9793	0.8788

The table shows the top-10 parameter combinations ranked by their scores (score = 0.7 * sensitivity + 0.3 * F1-score).

Supplementary Table 15 | Comparison table of SOTA models.

Method	SEN	SPE	PPV	NPV	F1-score	AUC
ResNet-50	0.7941	**0.9712**	**0.7714**	0.9747	**0.7826**	0.9623
ViT-Base	0.8529	0.9388	0.6304	0.9812	0.7250	0.9593
MambaOut	0.7941	**0.9712**	**0.7714**	0.9747	**0.7826**	0.9586
SigLipV2	0.8235	0.8489	0.4000	0.9752	0.5385	0.9034
MedViTV2	0.8824	0.8849	0.4839	0.9840	0.6250	0.9576
MedMamba	0.7941	0.8993	0.4909	0.9728	0.6067	0.9544
Query2label	0.8235	0.9424	0.6364	0.9776	0.7179	0.9471
Baseline	0.8235	0.9604	0.7179	0.9780	0.7671	0.9709
+ Multi-label head	0.9098	0.9037	0.6810	0.9780	0.7789	**0.9725**
+ Ensemble diagnosis(Ours)	**0.9118**	0.9317	0.6200	**0.9885**	0.7381	**0.9725**

This table compares the performance of SOTA models, including ResNet-50 and ViT-Base (classic SOTA models), MambaOut and SigLipV2 (SOTA classification models), MedViT-V2 and MedMamba (SOTA classification models in the medical field), and Query2Label (a SOTA multi-label classification model), alongside a baseline with enhancements. The baseline model is further improved with a multi-label head and ensemble diagnosis, where "+Ensemble diagnosis" represents our proposed method. Metrics include Sensitivity (SEN), Specificity (SPE), Positive Predictive Value (PPV), Negative Predictive Value (NPV), F1-score, and Area Under the Curve (AUC). The best result for each metric is highlighted in bold.

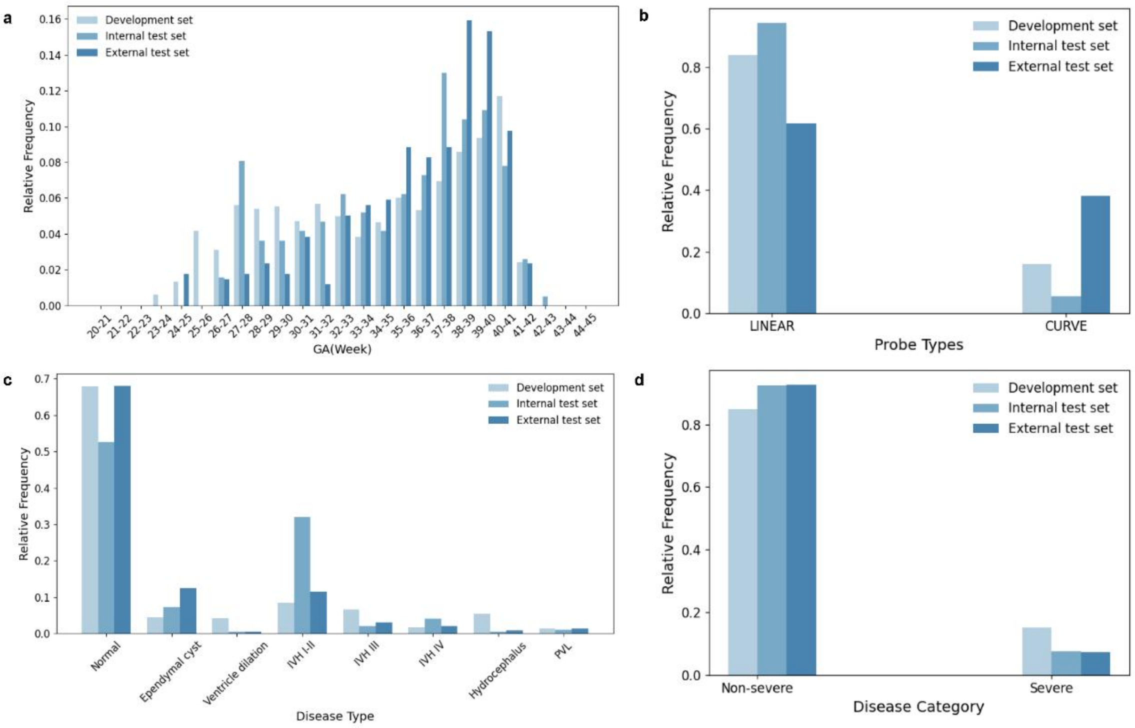

Supplementary Figure 1 | Statistical distribution of key clinical and technical parameters of different datasets. a The gestational age distribution, with the X-axis showing gestational age intervals (in weeks) and the Y-axis indicating the percentage distribution. **b** Probe type distribution. **c** Disease subtype distribution. **d** Brain injury severity classification.

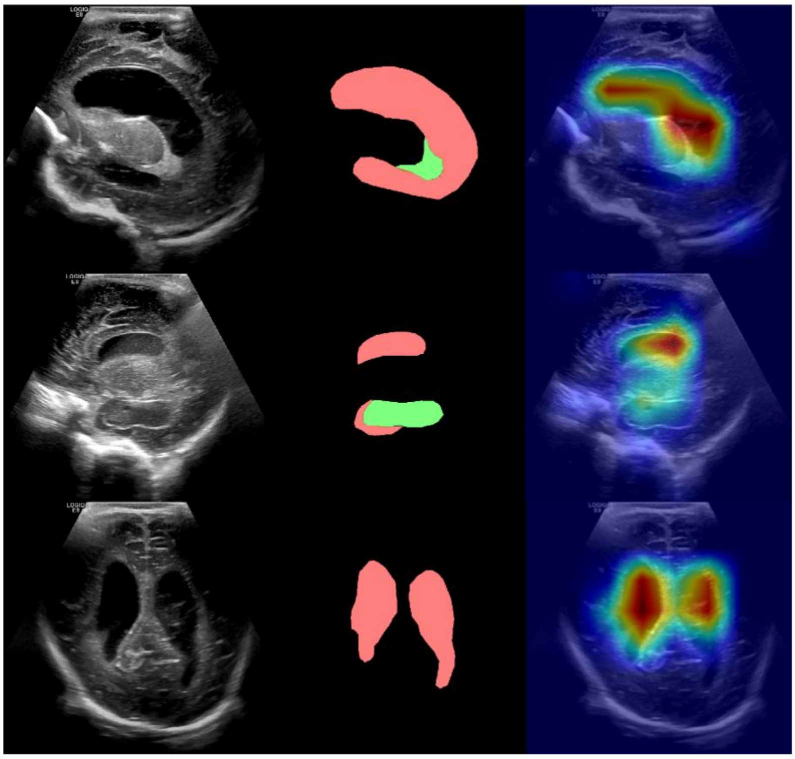

Supplementary Figure 2 | ROI annotated by the radiologist and the heatmaps. The first column shows the original images, the second column displays the ROIs of the ventricle (in red) and the choroid plexus or bleeding focus (in green). The third column presents the heatmaps.

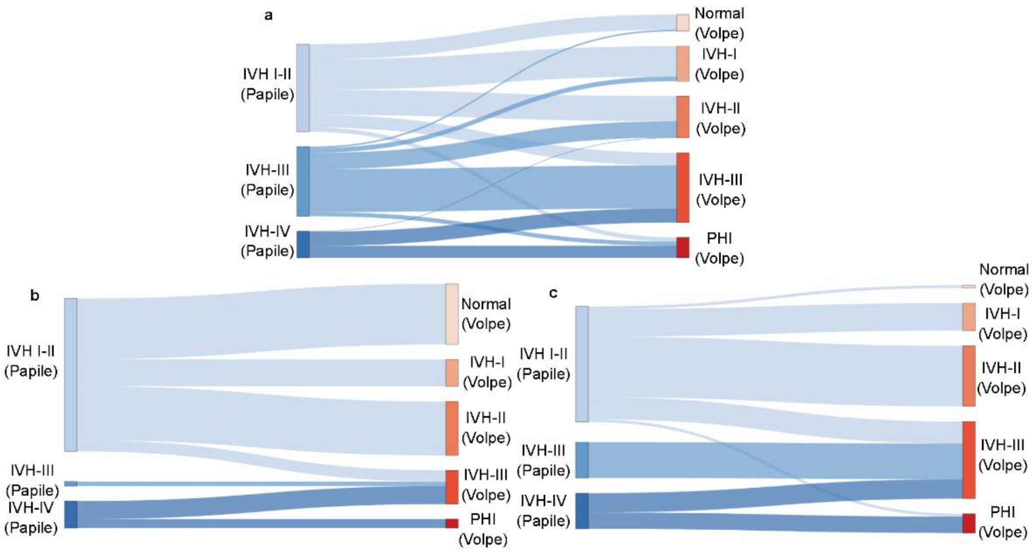

Supplementary Figure 3 | Reclassification of IVH Cases from Papile to Volpe Grading Systems across Datasets. a IVH grade redistribution in the development set. **b** IVH grade redistribution in the internal test set. **c** IVH grade redistribution in the external test set. All figures illustrate the distribution of IVH cases initially classified according to the Papile grading system (left) and their subsequent distribution after reclassification using the Volpe grading method (right) in the development set. Flow thickness represents the number of cases transitioning between grades. IVH, intraventricular hemorrhage; PHI, Periventricular Hemorrhagic Infarction.

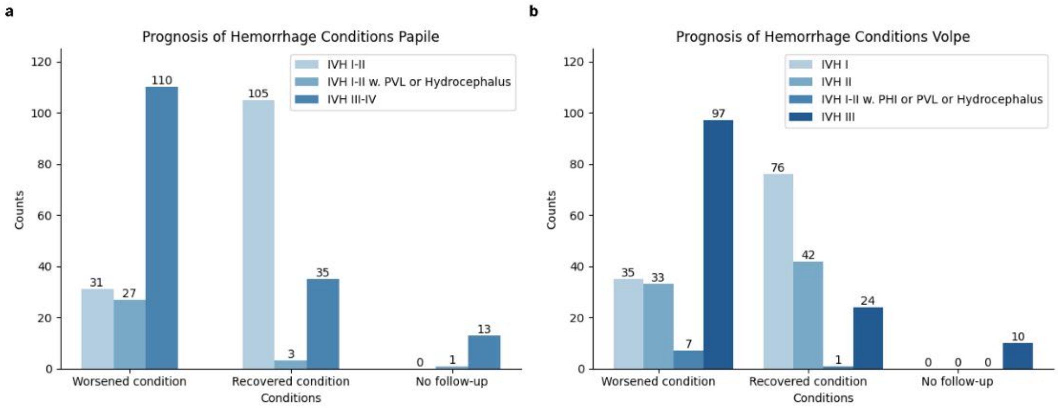

Supplementary Figure 4 | Prognostic Outcomes Under Two Grading Systems. a Prognostic outcomes under the Papile grading system. **b** Prognostic outcomes under the Volpe grading system. IVH, intraventricular hemorrhage; PHI, Periventricular Hemorrhagic Infarction; PVL, periventricular leukomalacia.

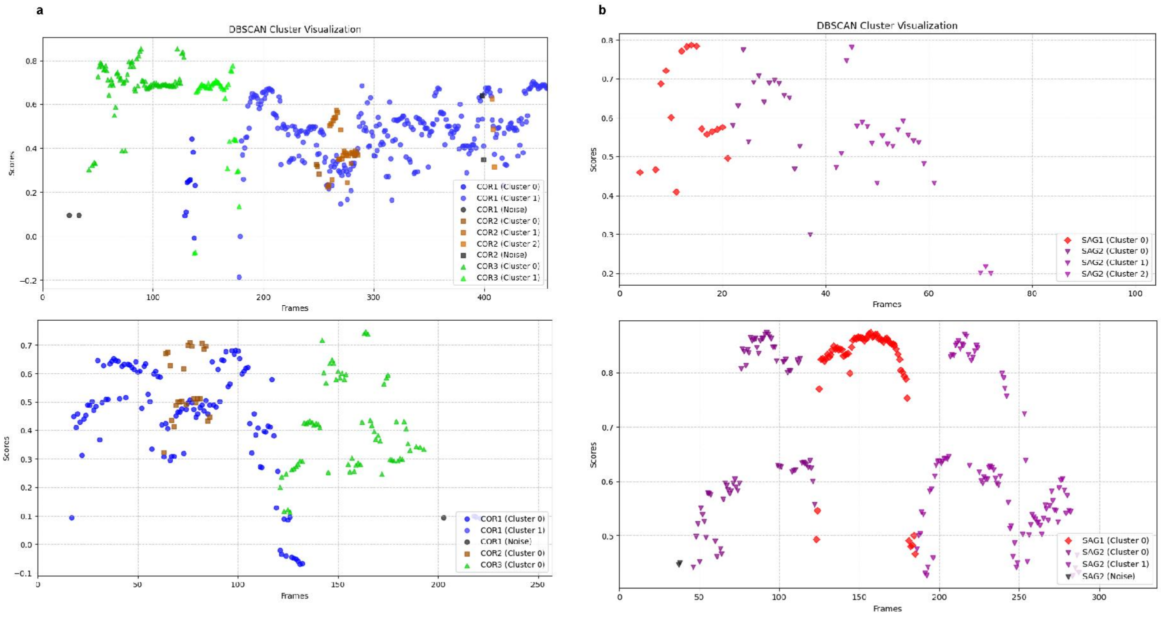

Supplementary Figure 5. DBSCAN clusters in CUS videos. a DBSCAN clusters in the coronal plane video. The brown cluster represents the cluster in the coronal plane slice of the lateral ventricle and third ventricle, while the green cluster represents the cluster in the body of the lateral ventricle. The direction of these two clusters helps determine the scanning direction of the ultrasound video, thereby eliminating the unnecessary clusters of the anterior horn of the lateral ventricle (blue). The images above and below show different scanning directions. **b** DBSCAN clusters in the sagittal plane video. The red cluster represents the mid-sagittal plane, and the purple cluster represents the para-sagittal plane. If two purple clusters are located on either side of the red cluster, we can distinguish these two clusters as frames from the left or right parasagittal plane. COR, coronal plane view; SAG, sagittal plane view.

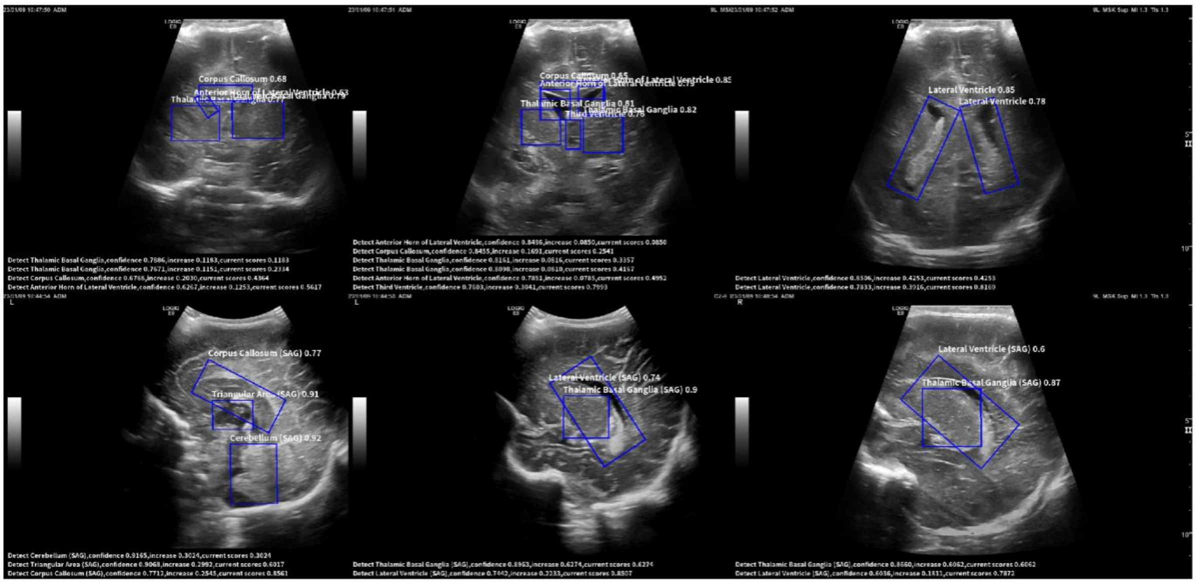

Supplementary Figure 6. Anatomical structures detection and candidate frame scoring. The bounding box was predicted by the model, and the label and value corresponded to the anatomical structure predicted by the model and the confidence score. The text below described the process of candidate frame scoring.

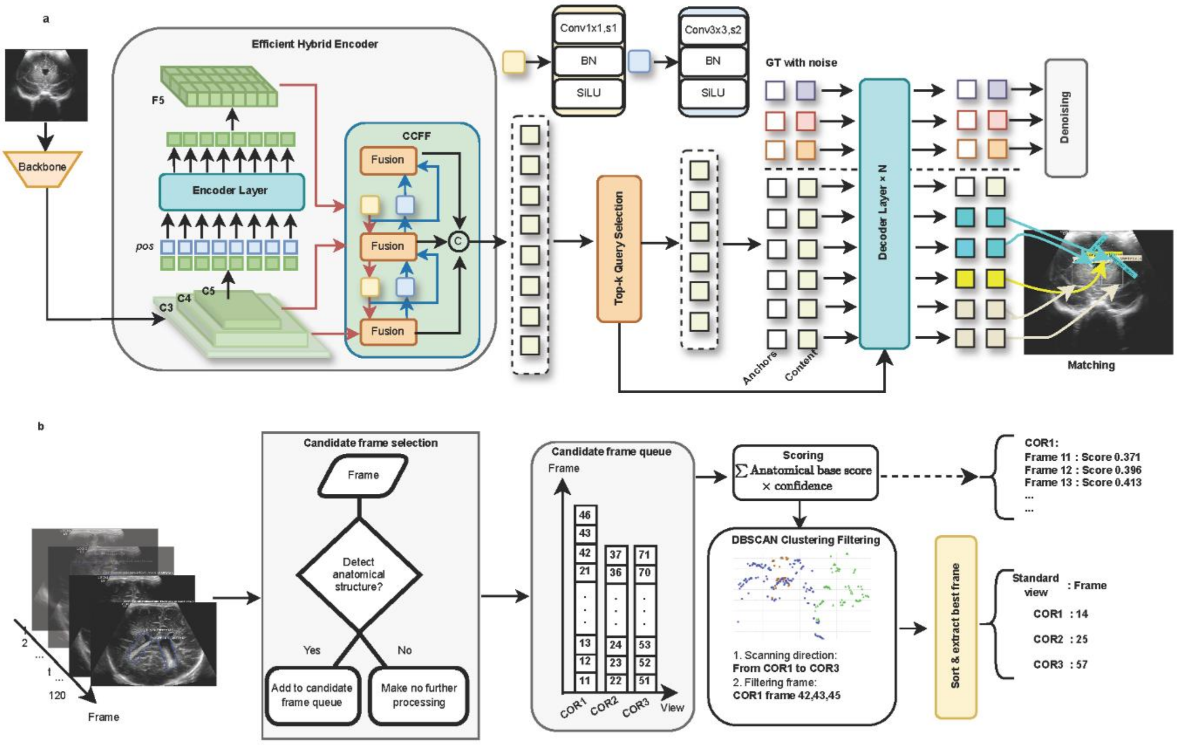

Supplementary Figure 7. The workflow of the view extraction module. a The architecture of the object detection model. **b** The workflow of the candidate frame scoring algorithm. COR1, Coronal plane anterior horn of the lateral ventricle view; COR2, Coronal plane third ventricle view; COR3, Coronal plane body of the lateral ventricle view. C and F represent features; BN, batch norm; SiLU, Sigmoid-weighted Linear Unit; COR, coronal plane view; GT, ground truth.

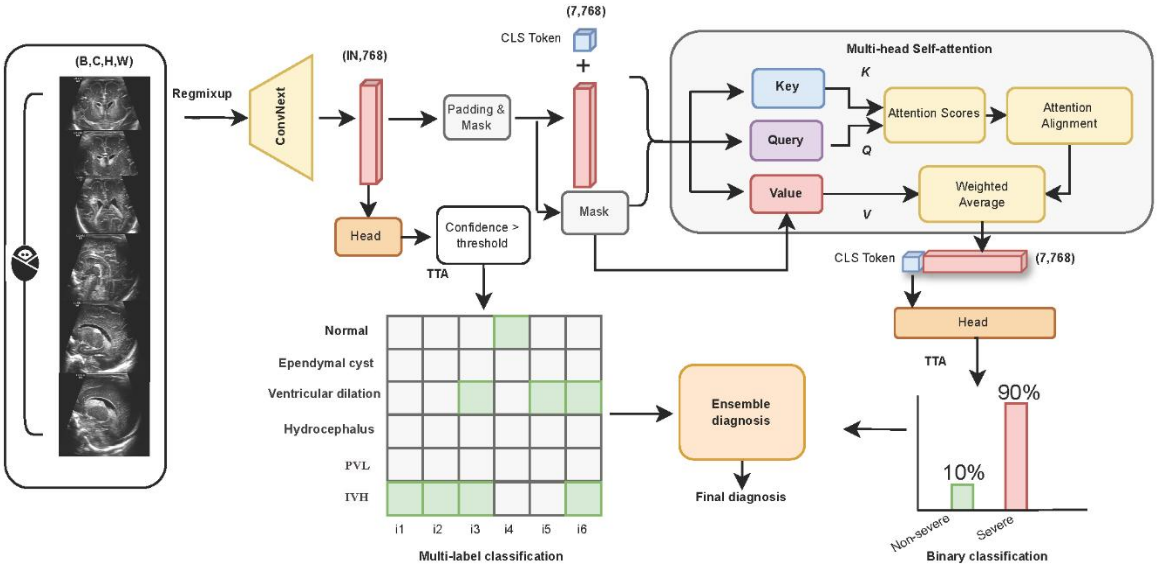

Supplementary Figure 8. The architecture of the diagnostic model. IN, number of images; TTA, test time augmentation; i1 to i6, CUS image1 to CUS image6; PVL, periventricular leukomalacia; IVH, intraventricular hemorrhage.
