# Supplementary material for: Deep learning approach for screening neonatal cerebral lesions on ultrasound in China
Source: Nat Commun. 2025 Aug 20;16:7778. doi: 10.1038/s41467-025-63096-9 (PMC12368173; doi:10.1038/s41467-025-63096-9)
Supplement: Supplementary file 2 — Reporting Summary [file 41467_2025_63096_MOESM2_ESM.pdf]

nature portfolio

Corresponding author(s): Luyao Zhou, Lei Liu, Dong Ni

Last updated by author(s): Apr 21, 2025

Reporting Summary

Nature Portfolio wishes to improve the reproducibility of the work that we publish. This form provides structure for consistency and transparency in reporting. For further information on Nature Portfolio policies, see our Editorial Policies and the Editorial Policy Checklist.

Statistics

For all statistical analyses, confirm that the following items are present in the figure legend, table legend, main text, or Methods section.

n/a	Confirmed	
☐	☒	The exact sample size (*n*) for each experimental group/condition, given as a discrete number and unit of measurement
☐	☒	A statement on whether measurements were taken from distinct samples or whether the same sample was measured repeatedly
☐	☒	The statistical test(s) used AND whether they are one- or two-sided<br>*Only common tests should be described solely by name; describe more complex techniques in the Methods section.*
☒	☐	A description of all covariates tested
☒	☐	A description of any assumptions or corrections, such as tests of normality and adjustment for multiple comparisons
☐	☒	A full description of the statistical parameters including central tendency (e.g. means) or other basic estimates (e.g. regression coefficient) AND variation (e.g. standard deviation) or associated estimates of uncertainty (e.g. confidence intervals)
☐	☒	For null hypothesis testing, the test statistic (e.g. *F*, *t*, *r*) with confidence intervals, effect sizes, degrees of freedom and *P* value noted<br>*Give P values as exact values whenever suitable.*
☒	☐	For Bayesian analysis, information on the choice of priors and Markov chain Monte Carlo settings
☒	☐	For hierarchical and complex designs, identification of the appropriate level for tests and full reporting of outcomes
☒	☐	Estimates of effect sizes (e.g. Cohen's *d*, Pearson's *r*), indicating how they were calculated

Our web collection on statistics for biologists contains articles on many of the points above.

Software and code

Policy information about availability of computer code

Data collection	We developed a internal labeling software for expert evaluation. Scripts for data collection and preparation were written in Python (3.10) using numpy (1.26.4), pandas (2.2.2).
Data analysis	We used Python and standard visualization and analysis toolkits (e.g., matplotlib and scipy) for data analysis.

For manuscripts utilizing custom algorithms or software that are central to the research but not yet described in published literature, software must be made available to editors and reviewers. We strongly encourage code deposition in a community repository (e.g. GitHub). See the Nature Portfolio guidelines for submitting code & software for further information.

Data

Policy information about availability of data

All manuscripts must include a data availability statement. This statement should provide the following information, where applicable:

- Accession codes, unique identifiers, or web links for publicly available datasets
- A description of any restrictions on data availability
- For clinical datasets or third party data, please ensure that the statement adheres to our policy

The data that support the findings of this study are available from the corresponding author upon reasonable request. Please contact zhouly6@mail.sysu.edu.cn for access.

Research involving human participants, their data, or biological material

Policy information about studies with human participants or human data. See also policy information about sex, gender (identity/presentation), and sexual orientation and race, ethnicity and racism.

Reporting on sex and gender	In this study, both male and female newborns were included. The sex of the newborns was determined based on biological attributes at birth. We have reported the number of male and female newborns in the study cohort in the Results section (see Table 1).
Reporting on race, ethnicity, or other socially relevant groupings	In this study, all newborns were recruited from hospitals in China, resulting in a relatively homogeneous population in terms of race and ethnicity.
Population characteristics	This study involved newborns recruited from hospitals in China. Key population characteristics of the newborns include their sex, gestational age, birth weight, and Apgar scores, which are detailed in the Results section and Table 1.<br>The clinical trials were conducted by radiologists from Shenzhen Children's Hospital. The radiologists involved ranged from junior to senior radiologists, with ages spanning 20 to 40 years. Their roles and experience levels were considered in the study design to ensure consistency in clinical assessments and data collection, as outlined in the Result section.
Recruitment	Newborns in this study were recruited from multiple hospitals in China. Eligible participants were required to meet the following specific clinical conditions: normal newborns, or those with intraventricular hemorrhage, ventricular dilatation, hydrocephalus, periventricular leukomalacia, or ependymal cysts, as confirmed by clinical assessments and imaging. The recruitment period spanned from January 2021 to the present.
Ethics oversight	This study was conducted as a retrospective and prospective analysis, approved by the institutional review boards of each participating institution. The retrospective development set and prospective internal test set were obtained from Shenzhen Children's Hospital (committee number 202312702), while the prospective external test set was collected from Guangzhou Panyu District Maternal and Child Health Care Hospital (IIT2023-17-02), Sichuan Provincial Maternity and Child Health Care Hospital (20240205-013), and Changsha Hospital for Maternal and Child Health Care (EC-20240102-09). The prospective section was registered at the Chinese Clinical Trial Registry (Registration number: ChiCTR2400079819).

Note that full information on the approval of the study protocol must also be provided in the manuscript.

Field-specific reporting

Please select the one below that is the best fit for your research. If you are not sure, read the appropriate sections before making your selection.

☒ Life sciences ☐ Behavioural & social sciences ☐ Ecological, evolutionary & environmental sciences

For a reference copy of the document with all sections, see nature.com/documents/nr-reporting-summary-flat.pdf

Life sciences study design

All studies must disclose on these points even when the disclosure is negative.

Sample size	The sample size for training and developing the AI model was determined based on an internal development set consisting of 8,757 neonatal CUS images, retrospectively collected from a single hospital, corresponding to 1,518 cases. For evaluation, the sample size of the test datasets was determined as follows: the internal video test set included 199 cases prospectively collected from the same hospital, and the external video test set comprised 356 cases prospectively collected from three other centers.
Data exclusions	The exclusion criteria for the CUS data were as follows: (1) incomplete CUS examinations; (2) extremely low-quality or unusable CUS data; (3) unclear diagnostic results; and (4) data from other ultrasound modalities, such as Color Doppler image.
Replication	Each evaluation task was conducted by several different level of radiologists from Shenzhen's Children hospital.
Randomization	Newborns' data (8,757 CUS images, 1,518 cases) from one hospital (Jan 2021–Jun 2022) were randomly split into training and testing sets, using stratified random sampling to maintain consistent proportions of clinical conditions. For evaluation, internal (199 cases) and external (356 cases) test sets were prospectively collected without randomization, focusing on natural condition distribution to assess generalizability. Additionally, 9 junior and 11 mid-level radiologists were randomly selected as evaluators to minimize experience bias.
Blinding	All investigators were completely blinded to the ground truth results.

Reporting for specific materials, systems and methods

We require information from authors about some types of materials, experimental systems and methods used in many studies. Here, indicate whether each material, system or method listed is relevant to your study. If you are not sure if a list item applies to your research, read the appropriate section before selecting a response.

Materials & experimental systems

n/a	Involved in the study
☒	☐ Antibodies
☒	☐ Eukaryotic cell lines
☒	☐ Palaeontology and archaeology
☒	☐ Animals and other organisms
☒	☐ Clinical data
☒	☐ Dual use research of concern
☒	☐ Plants

Methods

n/a	Involved in the study
☒	☐ ChIP-seq
☒	☐ Flow cytometry
☒	☐ MRI-based neuroimaging

Plants

Seed stocks

Report on the source of all seed stocks or other plant material used. If applicable, state the seed stock centre and catalogue number. If plant specimens were collected from the field, describe the collection location, date and sampling procedures.

Novel plant genotypes

Describe the methods by which all novel plant genotypes were produced. This includes those generated by transgenic approaches, gene editing, chemical/radiation-based mutagenesis and hybridization. For transgenic lines, describe the transformation method, the number of independent lines analyzed and the generation upon which experiments were performed. For gene-edited lines, describe the editor used, the endogenous sequence targeted for editing, the targeting guide RNA sequence (if applicable) and how the editor was applied.

Authentication

Describe any authentication procedures for each seed stock used or novel genotype generated. Describe any experiments used to assess the effect of a mutation and, where applicable, how potential secondary effects (e.g. second site T-DNA insertions, mosiacism, off-target gene editing) were examined.
